# Retraction Note: Circular RNA circCTNNA1 promotes colorectal cancer progression by sponging miR-149-5p and regulating FOXM1 expression

**DOI:** 10.1038/s41419-026-08452-3

**Published:** 2026-02-04

**Authors:** Pengju Chen, Yunfeng Yao, Nan Yang, Lifei Gong, Yuanyuan Kong, Aiwen Wu

**Affiliations:** 1 Key laboratory of Carcinogenesis and Translational Research (Ministry of Education), Department of Unit III & Ostomy Service, Gastrointestinal Cancer Center, Peking University Cancer Hospital & Institute, Beijing, 100142 China; 2 Department of Newborn Screening, Beijing Obstetrics and Gynecology Hospital, Capital Medical University, Beijing Maternal and Child Health Care Hospital, Beijing, 100026 China

Retraction Note to: *Cell Death & Disease* 10.1038/s41419-020-02757-7, published online 22 July 2020

The Editors have retracted this article. After publication, the authors contacted the journal to request a correction to replace the images presented in Figs. 1j, 2f, 5a and b, as well as Fig. 2j SW620 24h circCTNNA1 image, and Fig. 3a SW620 and SW480 sh-NC plots. The authors explained that the data were incorrectly selected for publication; however, further checks by the Publisher found highly similar features in the cell colony (Figs. 2j, 5a and b) and wound healing (Figs. 2j, 5g and h) images representing different groups, which could not be sufficiently addressed by this explanation.

The Editors therefore no longer have confidence in the presented data.

Pengju Chen does not agree with this retraction. The other authors have not responded to any correspondence from the editor or publisher about this retraction.

